# Operative technique in robotic pancreaticoduodenectomy (RPD) at University of Illinois at Chicago (UIC): 17 steps standardized technique: Lessons learned since the first worldwide RPD performed in the year 2001

**DOI:** 10.1007/s00464-018-6228-7

**Published:** 2018-05-15

**Authors:** Pier Cristoforo Giulianotti, Alberto Mangano, Roberto E. Bustos, Federico Gheza, Eduardo Fernandes, Mario A. Masrur, Antonio Gangemi, Francesco M. Bianco

**Affiliations:** Division of General, Minimally Invasive and Robotic Surgery, University of Illinois at Chicago, 840 S Wood Street, Suite 435 E, Clinical Sciences Building, Chicago, IL 60612 USA

**Keywords:** Robotic pancreaticoduodenectomy, Whipple procedure, Evidence-based surgery, Pancreatic surgery, Uncinate process, Pancreatic cancer

## Abstract

**Background:**

Minimally invasive pancreaticoduodenectomy (MIPD) was introduced in the attempt to improve the outcomes of the open approach. Laparoscopic pancreaticoduodenectomy (LPD) was first reported by Gagner and Pomp (Surg Endosc 8:408–410, 1994). Unfortunately, due to its complexity and technical demand, LPD never reached widespread popularity. Since it was first performed by P. C. Giulianotti in 2001, Robotic PD (RPD) has been gaining ground among surgeons. MIPD is included as a surgical option in the latest NCCN Guidelines. However, lack of surgical standardization, however, has limited the reproducibility of MIPD and made the acquisition of the technique by other surgeons difficult. We provide an accurate description of our standardized step-by-step RDP technique.

**Methods:**

We took advantage of our 15-year long experience and > 150 cases performed to provide a step-by-step guidance of our RPD standardized technique. The description includes practical “tips and tricks” to facilitate the learning curve and assist with the teaching/evaluation process.

**Results:**

17 surgical steps were identified as key components of the RPD procedure. The steps reflect the subdivision of the RPD into several parts which help to understand a strategy that takes into accounts specific anatomical landmarks and the demands of the robotic platform.

**Conclusions:**

Standardization is a key element of the learning curve of RPD. It can potentially provide consistent, reproducible results that can be more easily evaluated. Despite promising results, full acceptance of RPD as the ‘gold standard’ is still work in progress. Randomized-controlled trials with the application of a standardized technique are necessary to better define the role of RPD.

Pancreaticoduodenectomy (PD) is the only effective treatment of pancreatic head cancers [1–5]. Named after Allen Oldfather Whipple, the surgeon who popularized it in North America in 1935 [6], PD had been described by the Italian Surgeon Alessandro Codivilla in 1898 and subsequently by the German surgeon Walther Kausch [7]. PD is a complex technique due to the combination of the dissection in close proximity to vital vascular structures and the reconstruction which usually requires three challenging anastomoses [2, 8, 9]. Such procedural complexity may lead to considerable morbidity. Minimally invasive pancreaticoduodenectomy (MIPD) was introduced in the attempt to improve outcomes and to help minimize morbidity and mortality. Laparoscopic pancreaticoduodenectomy (LPD) was first reported by Gagner and Pomp in 1994 [10]. Unfortunately, due to its complexity, the technical challenges and the dexterity necessary during the reconstructive phase, LPD never reached widespread popularity [2, 11, 12]. Moreover, the long learning curve of LPD discouraged many surgeons from adopting this technique [11, 12].

More recently, the robotic surgery helped to overcome some of the limitations of the traditional laparoscopy.

The increased dexterity of the endowristed instruments, the superior quality of the motion with more degrees of freedom, the three-dimensional magnification of the camera, the software-mediated filtration of surgeon tremor, and the improved ergonomics allow unprecedented precision in the context of a long and accurate dissections required for pancreatic surgery [2, 13, 14].

The first robotic PD was performed by P. C. Giulianotti in 2001 and reported in 2003 [15]. After the initial experience, RPD gained popularity and, in some institutions, has become the standard of care for pancreatic head surgery. MIPD has been even included among the options in the latest NCCN Guidelines [16]. However, one of the hurdles to the wider adoption of the MIPD is the lack of surgical standardization. This has limited the reproducibility of MIPD and made the acquisition of the technique by other surgeons difficult.

With a 15-year long experience of > 150 cases, we present our standardized 17 steps Operative Technique for RPD (Table 1). This step-by-step technique includes relevant practical “tips and tricks” that may ease the learning curve and help surgeons who decide to approach this procedure.

## Operative technique of RPD

See Table 1.

**Table 1:** Step-by-step operative technique of RPD

Dissection
1. Gastrocolic ligament opening
2. Right colonic flexure mobilization
3. Kocher maneuver
4. Hilum exploration
5. Right gastric artery division
6. Right gastroepiploic artery division
7. Duodenum division
8. Cholecystectomy
9. Common bile duct transection
10. Gastroduodenal artery transection
11. First jejunal loop transection (at the Treitz ligament)
12. Pancreatic neck transection
13. Uncinate process dissection
Reconstruction
14. Pancreatojejunostomy or pancreatogastrostomy
15. Hepaticojejunostomy
16. Pylorojejunostomy or gastrojejunostomy
17. Specimen extraction and closure

UIC standardized 17 steps technique

## Trocar positioning and docking time

**Fig. 1:**
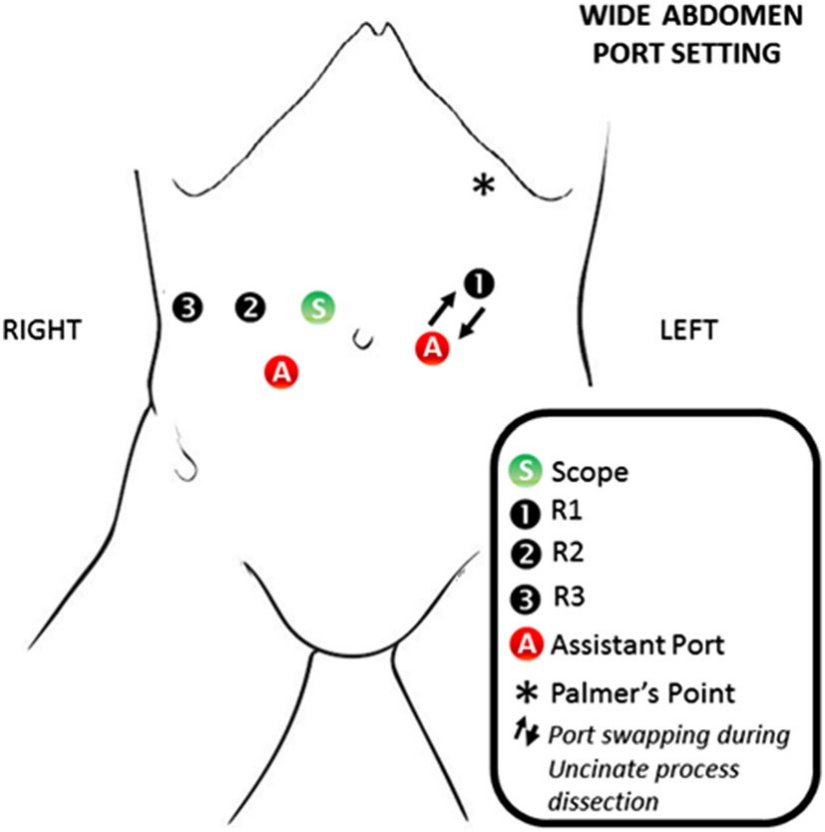
Port setting in case of wide abdomen

**Fig. 2:**
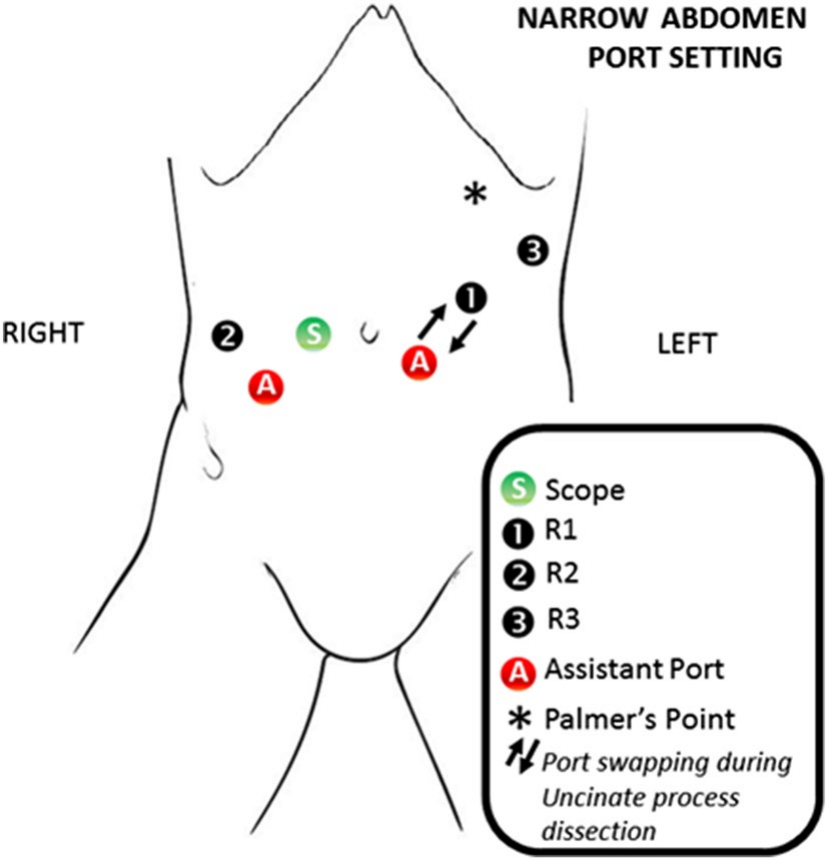
Port setting in case of narrow abdomen

The patient is placed on a bean bag in a supine 20-degree reverse Trendelenburg position with slight left-side tilt (arms tucked and lower limbs parted in the French position). The Assistant stands between the patient’s lower limbs. Pneumoperitoneum is induced with Veress needle at the Palmer’s point. A diagnostic laparoscopy is performed after the insertion of a 5-mm trocar in the left-upper quadrant. This is done to detect potential surgical contraindications (e.g., carcinomatosis, small liver metastases) and to place the other ports under vision. Standardization of the trocars placement is highly recommended, allowing for minor modifications according to the patient’s body conformation (Figs. 1, 2). For the Si-HD system, the cart is docked head on [15, 17, 18]. A 12-mm camera port is placed on the right pararectal line at the intersection with the transverse umbilical line. In this position, the scope provides a better visualization of the uncinate process, superior mesenteric vein (SMV), superior mesenteric artery (SMA), and portal vein. An additional 12-mm assistant port is positioned on the left side of the umbilicus. The first robotic arm (R1) is placed on the left side, 7–10 cm laterally to the assistant trocar. The second robotic arm (R2) is similarly placed on the right side. Another 5-mm assistant trocar is positioned in the area between the camera port and R2. According to the patient body structure, the 3rd arm (R3) trocar is positioned far lateral on either the right or the left side, depending on the size of the abdomen and the need of avoiding arms collisions. Our preference is to place the R3 on the right side, as it allows better retraction of the head of the pancreas during the uncinate process dissection [15, 17, 18]. When the Xi system is used, the trocar positioning is slightly different: four 8-mm robotic ports are positioned along a straight line and in a similar way to the one described for the Si-system. The patient cart is not necessarily docked from the patient’s head [15, 17, 18]. Just prior to docking, laparoscopic ultrasound is performed to rule out metastatic disease to the liver.

## Operative steps

### Dissection

#### Step 1. Gastrocolic ligament opening

A grasper is used via the R3 to lift the stomach cranially. The gastrocolic ligament is put under tension to identify the Bouchet lamina and to proceed to its dissection with the hook or other energy-based devices (harmonic shears or vessel sealer-Intuitive Surgical, Sunnyvale, CA). Access into the lesser sac allows inspection of the anterior pancreatic surface (body and neck) and the posterior gastric wall to exclude signs of local tumor spread/carcinosis. In case of pylorus-preserving PD (PPPD), the right gastroepiploic arcade must be identified and preserved in order to maintain adequate blood supply to the pylorus. Finally, the dissection of the gastrocolic ligament is carried out up to the short gastric vessels, which are preserved. Following the middle colic vessels, the confluence of the SMV with the portal vein at the neck of the pancreas is easily explored. The evaluation of this important venous confluence is part of the resectability assessment [15, 17, 18]. Laparoscopic US can be used in order to better assess the local extension of the disease and the vascular involvement.

#### Step 2. Right colic flexure mobilization

The takedown of the hepatic colonic flexure is carried out with the combined use of hook cautery (in R1) and bipolar forceps (in R2). The reverse Trendelenburg with left tilt rotation favors a downward retraction of the colon. A wide takedown of the hepatic colonic flexure is carried out up to the origin of the right colonic vessels medially and sometimes extended down to the cecum. This extended maneuver exposes the duodenum, SMV, and pancreatic head. An incomplete mobilization leads to difficult duodenal dissection and inadequate exposure of the ventral pancreatic surface [15, 17, 18]. Depending on the anatomy of the venous drainage, sometimes the right colic vein has to be divided. In fact, in case of high confluence or presence of a short Henle’s trunk, a complete exposure of the uncinate process cannot be achieved without interrupting this vein.

#### Step 3. Kocher maneuver

**Fig. 3:**
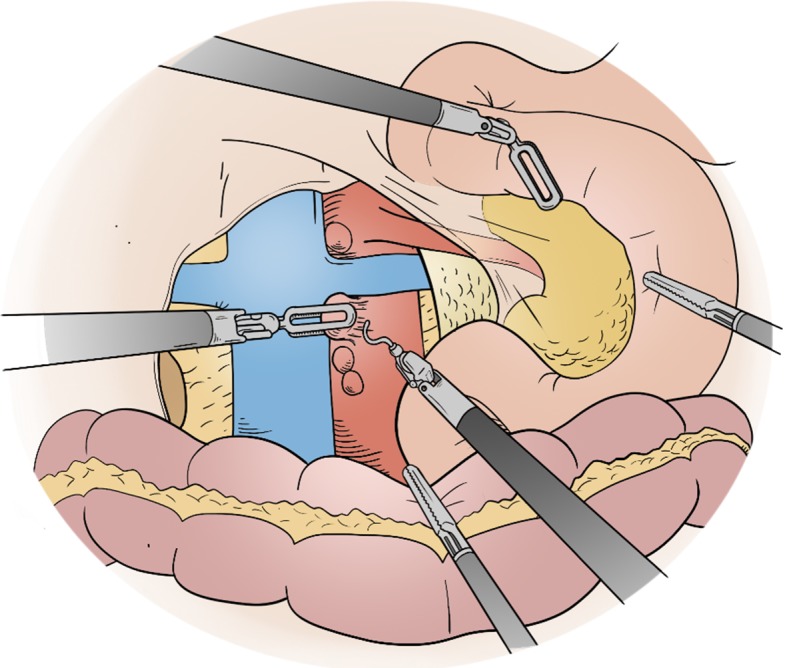
Extended Kocher maneuver with exposure of the left renal vein, aorta, and origin of the superior mesenteric artery

The Kocher maneuver (Fig. 3) must be extensive and requires an accurate stepwise approach. It can be sometimes difficult to be completed right at the beginning of the operation and may be achieved later on in the procedure following additional dissection of the pancreatic head. In any case, the Kocher maneuver needs to be wider compared to the open approach and has to be completed before attempting pancreatic transection. By doing so, the total detachment of the head of the pancreas off the retroperitoneal space allows a safer/easier uncinate process dissection. The left side of the aorta is the main landmark of the lateral edge of the dissection. One of the goals is the entire visualization of the posterior surface of the head of the pancreas. Other landmarks for the completion of the extended Kocher maneuver are left side of the aorta, left renal vein, and origin of the SMA. Enlarged lymph nodes in the interaortocaval space are harvested as part of the lymphadenectomy [15, 17, 18]. This step, as it is in the open approach, is part of the evaluation of resectability (i.e., the SMV/SMA encasement/involvement).

#### Step 4. Hepatic hilum exploration

During hepatic hilum exploration, the vascular anatomy must be carefully assessed, in particular, the possible presence of an accessory right hepatic artery originating from the SMA. If this is the case (up to 20%), the hilar dissection is more challenging. Depending on the size and relevance of the vessels (e.g., accessory vs totally replaced), as well as its relationship to the cancer, a decision regarding ligating, preserving, or reconstructing must be made. The dissection starts from the hilar plate exposing the right and left hepatic arteries and bile duct. The use of ICG fluorescence may be useful at this stage to confirm the anatomy of the biliary tree. Regional lymph nodes are sampled and sent for permanent pathology. Not all lymph nodes can be harvested en-block, and some of them can be retrieved station by station [15, 17, 18].

#### Step 5. Right gastric artery division

The right gastric artery is identified, skeletonized, ligated, and transected at the origin. This vessel may originate from the proper hepatic, common hepatic, or even left hepatic artery. The in-depth knowledge of such anatomic variations is essential to preserve enough pyloric blood supply while performing an accurate lymphadenectomy. Usually, the artery is ligated with 4/0 prolene suture [15, 17, 18].

#### Step 6. Right gastroepiploic artery division

The dissection around the of the right gastroepiploic artery is essential: the aim is to achieve a satisfactory nodal dissection maintaining a good blood supply of the pylorus. A vertical cranial lifting of the gastric antrum with R3 is beneficial to reach enough tension to identify the anatomy. The gastroepiploic artery is dissected out 1 cm from its origin and divided with vessel sealer or vascular stapler [15, 17, 18]. By doing so, the majority of the retropyloric lymph nodes remain attached en-block with the specimen (head of the pancreas).

#### Step 7. Duodenum division

The pyloric vascular supply and possibly the innervation have to be spared in case of PPPD. The vascular transection of the right gastroepiploic and right gastric artery has been already carried out. At this point, the posterior wall of the duodenum is dissected out until enough room is made to position the stapler and transect D 1, usually 1 cm distally to the pyloric sphincter. The stomach is then retracted to the left side. By doing this, a proper exposure of supra-pancreatic lymph nodes and the hepato-duodenal ligament is obtained [15, 17, 18].

#### Step 8. Gallbladder takedown

An anterograde cholecystectomy is carried out down to the cystic duct. In this way, an en-block resection is performed and the cystic duct is left attached to the distal common bile duct (CBD). Sutures or clips are applied to the cystic artery which is then divided with scissors [15, 17, 18].

#### Step 9. Common bile duct transection

The CBD is divided with cold-scissors if the duct is thin and small in size or by hook diathermy in case of a thick and fibrotic duct. The CBD division is usually cranial to the origin of the cystic duct and its proximal margin is sent for frozen section. The transection at this level allows adequate preservation of the proximal bile duct blood supply. A gentle bulldog is placed on the proximal CBD to prevent bile spillage in the surgical field. The distal portion of the bile duct is sutured and it remains attached to the specimen. The suture on the distal portion can be used to retract the CBD and get a better exposure of the portal vein [15, 17, 18].

#### Step 10. Gastroduodenal artery (GDA) transection

The GDA stump can represent a considerable source of post-operative complications and its transection it is not an easy step. Whenever there is enough room to apply it, vascular stapler is the safest approach. An accurate dissection and the division of some collaterals may be required in order to properly place the stapler. Suturing or ligating the GDA may be a backup plan in case not enough space is available. In any case, for the GDA vascular control, it is preferable not to use clips because of the risk of displacement. Attention should be made in identifying small tributaries of the portal vein (e.g., a variation of the right gastric vein). Any of these veins should be sutured and divided before approaching the gastroduodenal artery [15, 17, 18].

#### Step 11. First jejunal loop transection (at the Treitz ligament)

The duodenojejunal flexure division and the right-sided derotation of the duodenum must be carried out before performing the transection of the neck of the pancreas. The mesocolon is retracted cranially with the R3 in order to achieve exposure and enough tension on the suspension ligament. Monopolar hook or harmonic shears can be used for this maneuver. The aim is to extensively mobilize the fourth part of the duodenum. The first jejunal loop is divided with stapler. Of note, the more dissection is done at the Treitz, including ligation of some small jejunal vessels from the left side of the SMV, the easier will be to achieve complete derotation of the duodenojejunal flexure and the detachment of the uncinate process [15, 17, 18].

#### Step 12. Pancreatic neck transection

The neck of the pancreas is divided anteriorly to the portal vein. The safest way to carry out this step is to reach an optimal exposure of the inferior and superior margin of the pancreatic neck. On each side of the transection line, two 3/0 polypropylene stay sutures are placed on the inferior edge in order to retract and lift the pancreas and to control the bleeding from the pancreatic section line. The pancreatic neck is divided with the da Vinci Harmonic ACE™ Curved Shears (Intuitive Surgical, Inc). Increasing tension is applied on the stay sutures to retract the edges of the pancreas as the transection line advances. The classically described tunnel between pancreas and portal vein should be done only if it easy to do so. In case of inflammatory adhesions, the tunnel is developed progressively with the transection of the pancreatic parenchyma. This maneuver avoids to proceed safely within the periadventitial plane.

Following the parenchyma transection, the pancreatic duct is identified and cannulated with an endoluminal stent which is secured with 5/0 PDS (polydioxanone). If the pancreatic duct is inadvertently sealed by harmonic thermal lateral spread, it should be re-opened and stented. In order to have confirmation of free margins, a frozen section on the pancreatic duct is sent to pathology [15, 17, 18].

#### Step 13. The uncinate process dissection

This is the last and by far the most difficult step of the dissection. We believe that it is in this phase that the advantages of the robotic approach vs the open and laparoscopic ones are most evident. This is related to the overall superiority of the robotic platform when precise endowristed microsurgical skills are required. Also the R3 has a fundamental role in maintaining a stable retraction/exposure. All the venous branches originating from the SMV are selectively ligated. The dissection is conducted with the harmonic shears with application of prolene stitches for vessels of bigger caliber.

**Fig. 4:**
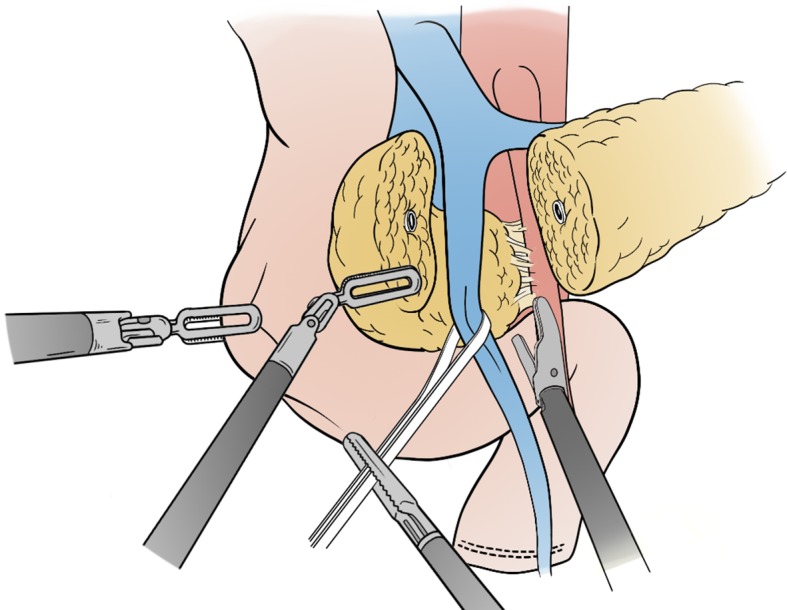
The venous “hanging maneuver” is performed by placing a vessel loop around the SMV

The venous “hanging maneuver” is performed by placing a vessel loop around the SMV (Fig. 4). This is done to get vascular control and retraction, and to allow a safe access to the space between the SMA and the SMV. The dissection proceeds in a caudocranial direction along the SMV. In robotic surgery, differently from open surgery (where the dissection can proceed in the opposite way), there are no alternatives to a bottom-up dissection of the uncinate process. Any branch originating from the SMV to the pancreas can be divided via Harmonic scalpel or between sutures. The angle of attack of the harmonic to this plane should be as close as possible to 90® degrees. This allows the best application of energy to the vessel in question. For this purpose, the instrument has to be switched in a port closer to the midline (usually through the 10–12 mm Assistant port with a telescopic maneuver). This because the harmonic shears are not articulating. The uncinate process dissection can vary slightly whether it is done for a malignant of a non-malignant lesion. In case of malignancy, the best technique is to expose the anterior aspect of the SMA just on the left side of the SMV. Using the hanging maneuver to retract the vein, the right side of the SMA can be accessed. The SMA can be progressively dissected and freed. The origin of the inferior pancreaticoduodenal artery is usually recognized and divided between 3/0 or 4/0 prolene sutures.

For benign or pre-malignant lesions, the dissection can be done with an energy base along the surgical plane of thin adipose/lymphatic tissue between the artery and the uncinate process of the pancreas. The harmonic shears can be very effective even in controlling the inferior pancreaticoduodenal artery if few precautions and tricks are put in place [15, 17, 18].

### Reconstruction

#### Creation of the Anastomoses

In the same way of the laparotomic approach, the reconstructive phase can vary according to (1) the pylorus preservation, (2) the pancreatic duct size, (3) the quality of the pancreatic parenchyma, and (4) the size of the bile duct. When a standard Whipple procedure is performed (with antrectomy), the pancreatogastrostomy can be more difficult and pancreatojejunostomy may become the best option. According to our standardized technique, a jejunal loop is passed where the D IV normally crosses the mesocolic root (retromesenteric route) and brought to the upper quadrants to reach the digestive reconstruction.

The main factors influencing the kind of pancreatic anastomosis to be performed are the pancreatic parenchyma texture and the pancreatic duct diameter. If the pancreatic tissue is fragile/soft or the duct is very small (< 3 mm), a transgastric pancreaticogastrostomy is the preferred option. Conversely, in case of firm/fibrotic pancreas and for duct > 4 mm a pancreaticojejunostomy is the best choice [15, 17, 18].

#### Step 14. Pancreaticogastro/jejunostomy

##### Pancreatojejunostomy

Multiple options are available to perform the pancreaticojejunostomy. The robotic approach guarantees the same technical level of sophistication of the laparotomic strategy, adding scaling effect and magnified view. In this way, the operative setting is very similar to the use of a surgical microscope platform.

An end-to-side duct to mucosa reconstruction is the preferred option when the pancreatic duct has a diameter ≥ 3 mm. The posterior capsule of the stump of the pancreas is anchored to the jejunal serosa via polypropylene sutures. A small opening in the jejunal mucosa is performed and the duct of the pancreas is anastomosed with PDS 4/0 or 5/0 interrupted stitches. Alternatively, two half-running sutures if the duct is ≥ 5 mm can be used. Inside the duct, a small stent is placed and secured with 5/0 PDS.

Another 4/0 polypropylene suture fixes the anterior aspect of the pancreatic capsule to the jejunum. Some additional interrupted stitches may be added to strengthen the suture.

If the capsule of the pancreas is fragile, a Blumgart’s technique can be used to anchor the pancreas to the jejunal loop [15, 17, 18].

##### Transgastric pancreatogastrostomy

**Fig. 5:**
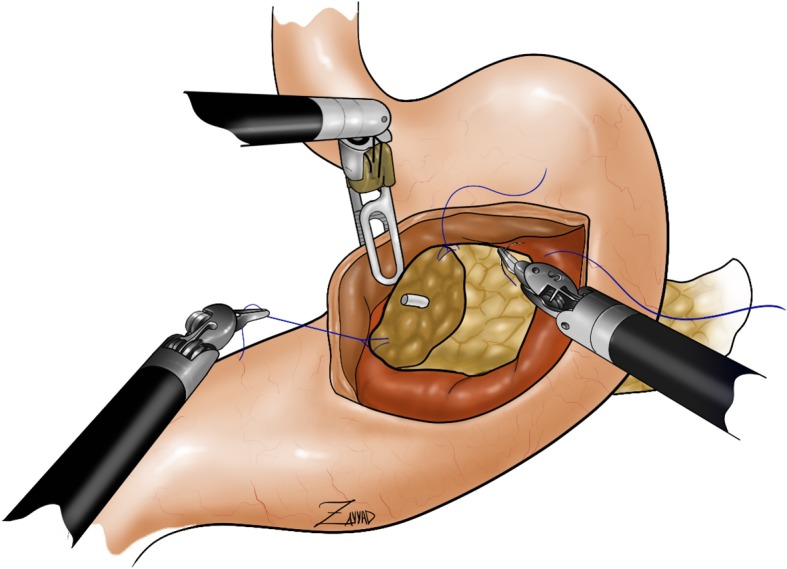
Pancreatogastro anastomosis. A longitudinal incision is performed on the stomach anteriorly; through an additional small opening on the posterior gastric wall, the pancreas is invaginated into the lumen. This maneuver is achieved by pulling the stay sutures on the pancreatic parenchyma through the anterior opening. Several 4/0 PDS interrupted stitches are placed between the pancreatic capsule and the gastric mucosa endoluminally

This is the preferred option for high-risk pancreatic parenchyma. The pancreatic stump needs to be freed for at least 4–5 cm. Any branch of the splenic vessels is divided between sutures, allowing mobilization of pancreatic stump. A longitudinal incision is performed on the stomach anteriorly; through an additional small opening on the posterior gastric wall, the pancreas is invaginated into the lumen. This maneuver is achieved by pulling the stay sutures on the pancreatic parenchyma through the anterior opening. Several 4/0 PDS interrupted stitches are placed between the pancreatic capsule and the gastric mucosa endoluminally (Fig. 5). Any minor bleeding should be meticulously controlled with additional stitches to achieve a perfect haemostasis. After the completion of the anastomosis, the closure of the anterior gastrotomy is performed with 3/0 PDS running suture. It seems that the pancreatic fistula following pancreatogastrostomies presents with less severity and morbidity because the pancreatic juice is inactivated. This technique, however, has also some disadvantages: (1) requires more preparation of the pancreatic stump; (2) has a higher risk of post-operative bleeding; (3) has greater long-term pancreatic atrophy risk leading to both endocrine and exocrine secretions [15, 17, 18].

#### Step 15. Hepaticojejunostomy (biliary reconstruction)

The hepaticojejunostomy is carried out in an end-to-side fashion. Usually a 4/0 or 5/0 PDS running suture is used for the posterior raw, and interrupted stitches are placed in the anterior one. The reconstruction of the anterior layer is performed starting from right side of the duct, placing the stitches 1 mm apart. A clip is positioned after placement of each stitch. This temporarily secures the tails of the sutures and prevent their displacement. After all stitches are placed, they can be tied individually. In case of a very dilated and thick bile duct, a running suture may be used for the anterior row as well [15, 17, 18].

#### Step 16. Pylorus/gastrojejunostomy (duodenojejunal reconstruction)

When a PPPD is performed, this anastomosis is placed on the same loop of the biliary anastomosis 50–60 cm distally to it. An end-to-side anastomosis is carried out using two 3/0 PDS running sutures. Some polypropylene interrupted stitches are commonly placed to strengthen the corners and the anterior wall. Indocyanine green (ICG)-enhanced fluorescence test can be performed to check adequate microvascular blood supply to the duodenal margins before making the anastomoses [15, 17, 18]. It is sometimes necessary to shorten the length of the duodenal stump closer to the pylorus to get viable tissue. A suboptimal perfusion may be responsible of edema and delayed gastric empting.

#### Step 17. Extraction of the specimen and closure

The specimen is placed in an endobag and extracted through a small Pfannenstiel incision. Alternative extraction sites may be considered in case of previous abdominal surgery. The specimen retrieval can be performed before starting the reconstruction or at the very end of the procedure (if there is no needed for tissue banking).

Two drains are usually placed: one close to the pancreatic anastomosis and the other one near the biliary anastomosis [15, 17, 18].

## Discussion

Recent scientific debate surrounding minimally invasive versus open pancreatic surgery focused on perioperative complications and oncological outcomes. After an initial period of skepticism, robotic pancreatic surgery has been embraced by some surgeons and MIPD is even included among the options in the latest NCCN Guidelines (Version 3.2017 Pancreatic Adenocarcinoma NCCN Evidence Blocks™) [16]. Despite this advance, the MIPD is still under evaluation [19, 20]. There is a limited number of centers specialized in pancreatic minimally invasive surgery, and well-powered randomized-controlled trials are difficult to put in place. This standardized description of the operative technique is the first attempt to produce an accurate step-by-step guide of RDP. The advantages of such a standardization are as follows:

1. To ease the learning curve of other surgeons approaching this robotic technique.
2. For each step of the procedure to facilitate homogeneous and reproducible results among different centers/surgeons [17, 21].

Our technique stems from a 15 years of experience and > 150 RPDs. The number of steps is arbitrary, but it reflects a careful consideration of the different anatomical fields encountered during surgery. The steps also reflect specific challenges encountered at certain points in the procedure, which are better faced when frame-worked within a specific step. This subdivision also allows for a better quantitative and qualitative assessment of the learning process and skills acquisition [17].The learning curve of the RPD is long and difficult to climb in a cost-effective manner. Suggested minimum numbers of cases required for surgical proficiency are 50 cases for laparoscopic and between 33 and 80 cases for the robotic PD [22–24]. The stepwise approach can also improve surgical training at fellow level. Adding to the potentials of the dual console in robotic surgical training [25], the stepwise approach can make it easier for a learner to grasp a complex procedure.

The stepwise approach facilitates also the teaching process focusing on specific anatomical landmarks of every step. Moreover, the step-by-step approach facilitates also (a) the interaction with the bedside team; (b) how to prevent dangerous situations or potentially life-threatening complications. All the aforementioned points are better addressed focusing on the single specific step of the procedure.

In general, RPD is a procedure that requires excellent laparoscopic and robotic surgical skills. However, there are specific steps that need more time and experience to be mastered:

1. The execution of an extended Kocher maneuver.
  The reason of such an extended kocherization is twofold: (a) functional to successful and safe completion of uncinate process dissection; (b) fundamental for the assessment of the resectability which is one of the weaknesses of the MIPD.
2. Uncinate process dissection.
  In open surgery, many authors perform a cranial–caudal approach. On the contrary, during MIPD a caudal–cranial technique has to be preferably used and associated with the venous hanging maneuver.
3. Complex reconstruction with a small pancreatic duct and/or a soft pancreas.
  This step requires considerable experience to be performed proficiently.

A flawless technique is necessary to decrease the chances of related complications. With the standardized approach, operative time can be shorter; and a smoother/faster learning curve may potentially lead to a progressive reduction in morbidity and a progressive reduction in morbidity. Despite promising results, full acceptance of RPD is hindered by the absence of randomized-controlled trials, by the paucity of specialized centers, and by a high variability of surgical techniques and perioperative management described in published studies [26, 27]. A long pathway ahead awaits RPD before it becomes the gold standard for pancreatic head cancers. However, significant steps have been made since its inception, and the introduction of more sophisticated robotic platforms will foster the process even further.

Whenever a new technique or surgical technology is introduced in clinical context, its data-driven validation is essential [28] Randomized-controlled trials with a standardized technique (followed by a meta-analytic assessment) are the ultimate tool [28], and multicenter efforts should be made to achieve this goal.
